# Decomposition of recalcitrant carbon under experimental warming in boreal forest

**DOI:** 10.1371/journal.pone.0179674

**Published:** 2017-06-16

**Authors:** Adriana L. Romero-Olivares, Steven D. Allison, Kathleen K. Treseder

**Affiliations:** 1 Department of Ecology and Evolutionary Biology, University of California Irvine, Irvine, CA, United States of America; 2 Department of Earth System Science, University of California Irvine, Irvine, CA, United States of America; Tennessee State University, UNITED STATES

## Abstract

Over the long term, soil carbon (C) storage is partly determined by decomposition rate of carbon that is slow to decompose (i.e., recalcitrant C). According to thermodynamic theory, decomposition rates of recalcitrant C might differ from those of non-recalcitrant C in their sensitivities to global warming. We decomposed leaf litter in a warming experiment in Alaskan boreal forest, and measured mass loss of recalcitrant C (lignin) vs. non-recalcitrant C (cellulose, hemicellulose, and sugars) throughout 16 months. We found that these C fractions responded differently to warming. Specifically, after one year of decomposition, the ratio of recalcitrant C to non-recalcitrant C remaining in litter declined in the warmed plots compared to control. Consistent with this pattern, potential activities of enzymes targeting recalcitrant C increased with warming, relative to those targeting non-recalcitrant C. Even so, mass loss of individual C fractions showed that non-recalcitrant C is preferentially decomposed under control conditions whereas recalcitrant C losses remain unchanged between control and warmed plots. Moreover, overall mass loss was greater under control conditions. Our results imply that direct warming effects, as well as indirect warming effects (e.g. drying), may serve to maintain decomposition rates of recalcitrant C compared to non-recalcitrant C despite negative effects on overall decomposition.

## Introduction

High-latitude soils store approximately 510 Pg of C, primarily owing to the buildup of recalcitrant C [1], such as lignin. Much of this soil C has decomposition rates of years to centuries, due to its complex chemical structure and exposure to cold temperatures [2–4]. Global warming is particularly rapid at high-latitudes [5–7] and as a result, decomposition of soil C may increase, reducing high-latitude C stocks [3]. If so, the CO_2_ released from these soils might form a positive feedback to global warming [3,7–10].

Moreover, recalcitrant C decomposition may be especially sensitive to temperature [3,11]. This idea is based on the theories of collision and enzyme kinetics, which imply that temperature sensitivity of decomposition is positively related to the complexity of the substrate [3,11]. In other words, the breakdown of complex recalcitrant C requires more enzymatic steps with higher activation energies [11–13]. Accordingly, Davidson and Janssens (2006) predicted that 2°C warming would increase decomposition of recalcitrant C by 21%, compared to only a 10% increase for non-recalcitrant C. The consequences of this difference should be exacerbated at high-latitudes, where a 2–5°C warming is predicted by the end of this century [7].

In addition to the direct kinetic effects of warming on decomposition, warming may also select for microbial communities that preferentially degrade recalcitrant C [14,15]. For example, some filamentous fungi are less tolerant to cold stress compared to yeasts [16] and might proliferate under warming; in addition, some of these filamentous fungi are better at decomposing recalcitrant C [17]. Variation in microbial breakdown of recalcitrant C compounds can disproportionately influence long-term C storage in soils [3,18]. However, changes in decomposition of recalcitrant C, specifically, are rarely assessed in field-based warming experiments [19] and are thus challenging to predict. In this warming experiment, Treseder and collaborators [17] reported that warming induced a shift in fungal community composition toward taxa that could break down recalcitrant C. Was there a concomitant shift in C use toward recalcitrant C under warming? Here, we tested this question by examining decomposition of recalcitrant C versus non-recalcitrant C in plant litter under experimental warming.

According to Hudson [20], lignin is more recalcitrant than soluble sugars, hemicellulose, and cellulose due to its complex chemical structure. Although there is conflicting evidence regarding long-term lignin stability [21] and the definition of recalcitrance [22], in this paper we refer to lignin as recalcitrant C. We grouped the less chemically-complex soluble sugars, hemicellulose, and cellulose, as non-recalcitrant C. We hypothesized that ratios of recalcitrant C to non-recalcitrant C (i.e. lignin: soluble sugars + hemicellulose + cellulose) remaining in decomposed litter would be lower in the warming treatment than in controls. In addition, we predicted that extracellular enzymes produced by microbes would target recalcitrant C (relative to non-recalcitrant C) more under warming.

## Methods

### Field site

The study area was located in a mature black spruce (*Picea mariana*) forest on the Fort Greely military base near Delta Junction, Alaska, USA (63°55’N, 145°44’W) [23]. At this site, the vegetation was dominated by black spruce with an understory of shrubs, mosses, and lichens. The climate was cold and dry, with approximately 303 mm y^-1^ of precipitation and a mean annual temperature of -2°C. The growing season extends from mid-May to mid-September.

### Warming experiment

In July 2005, a warming experiment was established as described in Allison & Treseder (2008). Five pairs of 2.5 x 2.5 m plots were marked in a 1 km^2^ area; one plot from each pair was assigned as the treatment while the other one was assigned as the control. Control plots were left under ambient conditions while treatment plots were warmed passively with greenhouses (closed-top chambers). Gutters and tubing were installed to direct precipitation into the greenhouses during the growing season. Greenhouses were left in place but the top plastic panels of the greenhouses were removed in mid-September and re-installed in mid-May to allow snowfall to enter the warmed plots. The warming treatment increased air temperature on average by 1.6°C, and soil temperature (5 cm depth) by 0.5°C. In addition, the warming treatment reduced soil moisture by 22% on average due to higher evapotranspiration [24].

### Litterbag experiment

On May 22, 2013, the warming experiment had been ongoing for eight years. On this date, we detached brown senescent spruce needles of living black spruce trees near the experimental plots by shaking branches lightly and/or by touching them and collecting the fallen needles in a plastic bag. Immediately after collection, we filled litterbags (10 x 10 cm, 1 mm mesh of nylon covered with a layer of 1 mm fiberglass mesh) with 2 g of spruce needles. We deployed four sets of two litterbags in the forest floor of each plot and took five subsamples of spruce needles for initial litter chemistry analysis.

We retrieved a set of litterbags after 1, 2, 12, and 16 months. We combined the contents of each of the two litter bags within each plot. Therefore, for each sampling time point we had five samples from control plots and five from warmed plots (n = 5). In the lab, we determined total fresh weight, then separated ~0.6 g for extracellular enzyme activity (EEA) measurements and ~0.5 g for litter chemistry. The EEA subsample was stored at -80°C, and the litter chemistry subsample was stored at -20°C. In addition, we used ~0.5 g from the first collection for fungal DNA sequencing; these findings are reported in Treseder et al. [17]. We determined fresh weight of the remaining litter, and dried it at 70°C for two days to obtain percent dry weight. We calculated litter mass remaining as the product of total fresh weight and fraction dry weight.

We have permission from Ft. Greely to work in this study location. No specific permissions were required for the activities in the current study. All samplings took place within public space in the forest and no military areas were accessed. No endangered or protected species were involved in this research.

### Litter chemistry

Litter samples were air dried for 48 hours and ground for 1 min in a Spex SamplePrep 8000D mixer/mill (Spex SamplePrep LLC, New Jersey) using stainless steel vials and grinding balls. To determine concentration of non-recalcitrant C (i.e. soluble sugars, cellulose, and hemicellulose) and recalcitrant C (i.e. lignin), we processed litter samples following Talbot et al. [25]. Samples were fractionated following the International Association of Analytical Communities (AOAC International) official Uppsala method [26]. In all cases, we performed triplicate measurements of each of the five replicates. For each date and each assay, we used two blanks to account for background absorbance.

#### Soluble sugars

First, we extracted and discarded lipids, waxes, and pigments with 100% petroleum ether. Next, we extracted soluble sugars with 80% ethanol and removed starch by α-amylase digestion. The starch-less fraction was used to determine glucose concentration by the phenol-sulfuric acid method [27]. We then washed the samples with 95% ethanol and 100% acetone, followed by drying at 70°C for 48 hours to obtain a lipid- and sugar-free fraction to quantify lignin, hemicellulose, and cellulose concentration.

#### Cellulose

We determined cellulose by the Updegraff method [28]. This method consists of the removal of hemicellulose and lignin and the extraction of cellulose with acetic acid/nitric acid followed by solubilization of cellulose in 67% sulfuric acid. We quantified cellulose concentrations via the Anthrone reaction in sulfuric acid at 100°C in a water bath, and measured absorbance at 620 nm. We used crystalline cellulose (MP biomedical cat. 02191499) as a standard.

#### Hemicellulose

Similarly, we measured hemicellulose in the acetic/nitric extracts of the Updegraff method by Hansen & Møller [29] with modifications following Aravantinos-Zafiris et al. [30]. We used a mixture of 10:7.5:7.5:7.5:7.5:5:5 of glucose, xylose, arabinose, mannose, galactose, fucose, and rhamnose as a standard. We quantified sugar concentrations by measuring absorbance at 630 nm.

#### Lignin

Finally, we determined total lignin by the acetyl bromide method [31], in which lignin is solubilized in 1:4 acetyl bromide:acetic acid solution and quantified by measuring absorbance at 280 nm. We used alkali lignin (Sigma cat. 370959) as a standard.

### Extracellular enzymes

To assess decomposer investment in recalcitrant C degradation under warming, we assayed the activities of four extracellular enzymes involved in decomposing different types of C, as previously described [32,33]. We performed this assay on litter that had decomposed 12 months, since this was the timepoint with the highest enzyme activity (marginal enzyme activity was detected on previous dates and thus, not included in our analyses). Using pyrogallol as the substrate, we assayed polyphenol oxidase (PPO) that degrades lignin as an index of the enzymatic potential to decompose recalcitrant C. As an index of the enzymatic potential to degrade non-recalcitrant C we assayed cellobiohydrolase (CBH) that targets cellulose, β-xylosidase (BX) that targets xylose ―a component of hemicellulose― and β-glucosidase (BG) that catalyzes the hydrolysis of glycosidic bonds in later steps of cellulose degradation (soluble sugars). Litter samples were homogenized in 50 mM maleate buffer, pH 6.0, and pipetted into microplates. We measured enzyme Vmax (nmol h^-1^ g^-1^ dry litter) at 4, 10, 16, 22, 28, and 34°C either colorimetrically (PPO), or fluorimetrically (CBH, BX, BG) on a microplate reader. Enzyme Vmax values were obtained by fitting the Michaelis-Menten equation to reaction velocities as a function of substrate concentration using the non-linear least squares (nls) method in R. Enzymes were assayed across a range of temperatures and substrate concentrations because these measurements were conducted as part of a separate study on the temperature sensitivity of enzyme Vmax and Km parameters. Fitted Vmax values were normalized to an overall mean of 1 for PPO and 1/3 for each of the other three enzymes. With the normalized values, we calculated activity ratios as PPO/(BG+BX+CBH) such that ratios >1 indicate greater relative investment in recalcitrant C degradation. Because there were no interactions between incubation temperature and the field warming treatment, activity ratios were averaged across incubation temperatures to obtain a single ratio for each experimental plot.

### Statistical analysis

To test our hypothesis, we conducted repeated measures analyses of variance (ANOVAs). Our dependent variable was the ratio of recalcitrant to non-recalcitrant C remaining, and the independent variable was warming treatment. Sampling date was the temporal factor. We conducted the same tests for mass remaining of each C fraction and for overall mass loss. For statistically significant ANOVAs, we followed up with post hoc t-tests to compare means within each sampling date.

For our prediction that the EEA activity ratios of recalcitrant to non-recalcitrant C should increase with warming, we performed a mixed-model ANOVA with block as a random factor. Our independent variable was warming treatment, and our dependent variable was the EEA activity ratio of recalcitrant to non-recalcitrant C. We log-transformed EEA activity ratios to meet assumptions of normality. All data were analyzed using R software [34].

## Results

**Fig 1:**
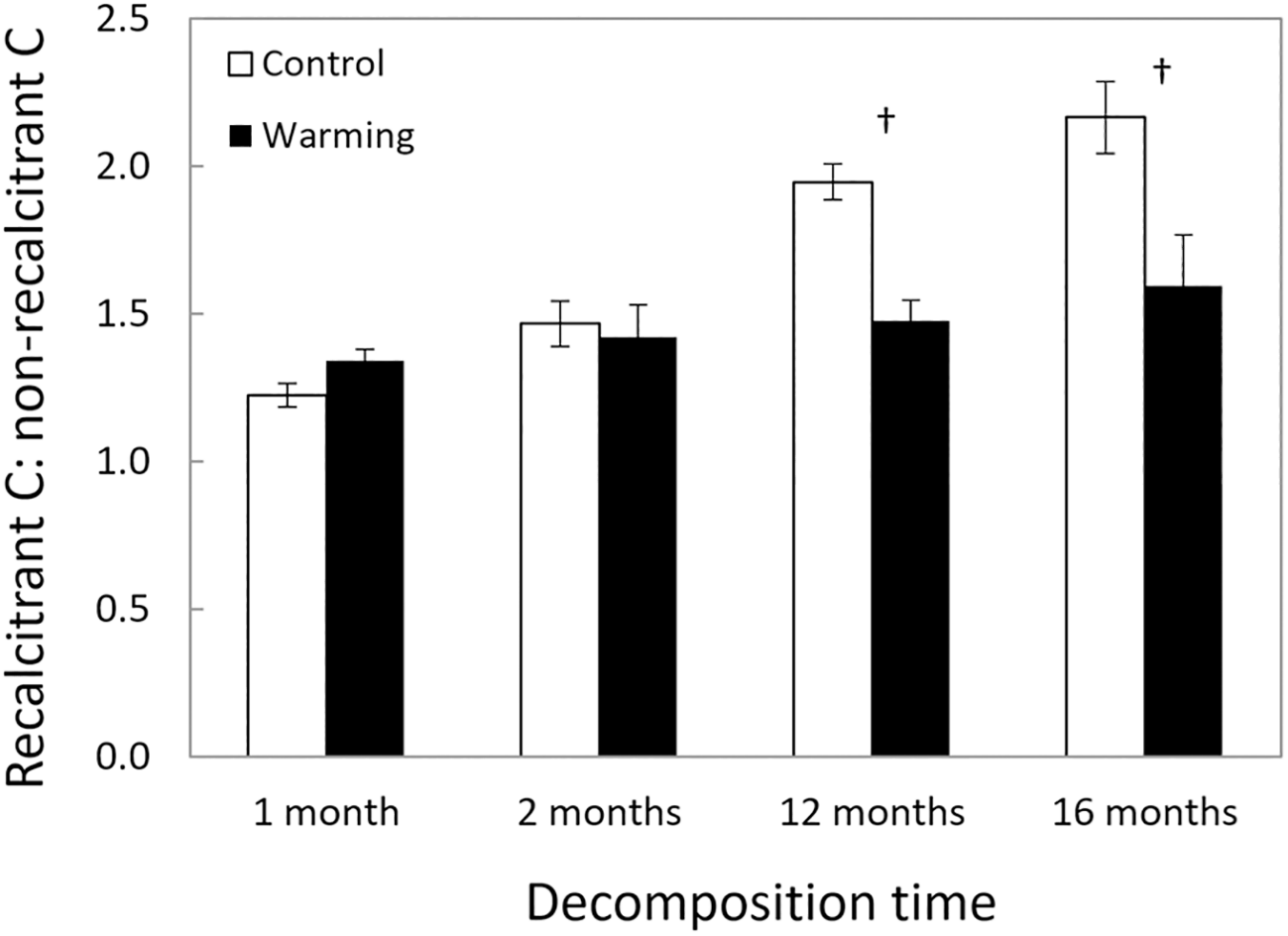
Mass remaining of recalcitrant (lignin) to non-recalcitrant (cellulose, hemicellulose, and soluble sugars) C over time. Across sampling times, the ratio was significantly lower in the warming treatment (P = 0.032), but there was a significant interaction between treatment and time (P = 0.046). Data are means ± SE, with n = 5 plots. †P < 0.10 for sampling date.

**Fig 2:**
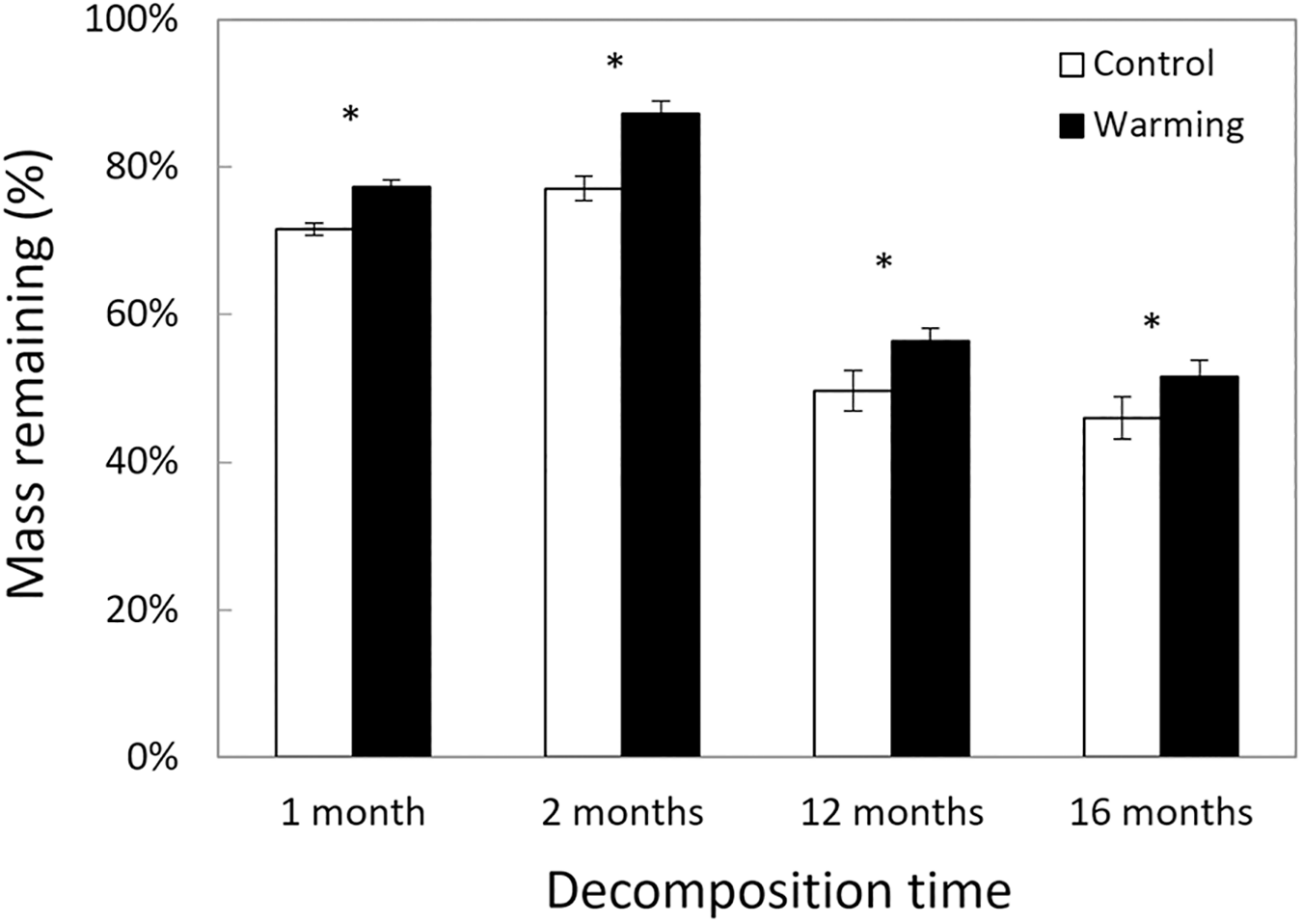
Percentage total mass remaining in spruce needles over time. Decomposition was significantly slower in the warming treatment compared to the control (P = 0.009). Data are means ± SE, with n = 5 plots. *P < 0.05 for sampling date.

The ratio of recalcitrant C to non-recalcitrant C remaining in decomposing litter was significantly lower in the warmed treatments than in the controls, but only after 12 months of decomposition (Fig 1, warming x date interaction, F_3,24_ = 3.102, P = 0.046). Interestingly, across all sampling dates, significantly more litter mass remained in the warmed treatment than in the control (Fig 2, F_1,8_ = 11.91, P = 0.009) and there was no significant interaction with sampling date (F_3,24_ = 0.98, P = 0.419).

**Fig 3:**
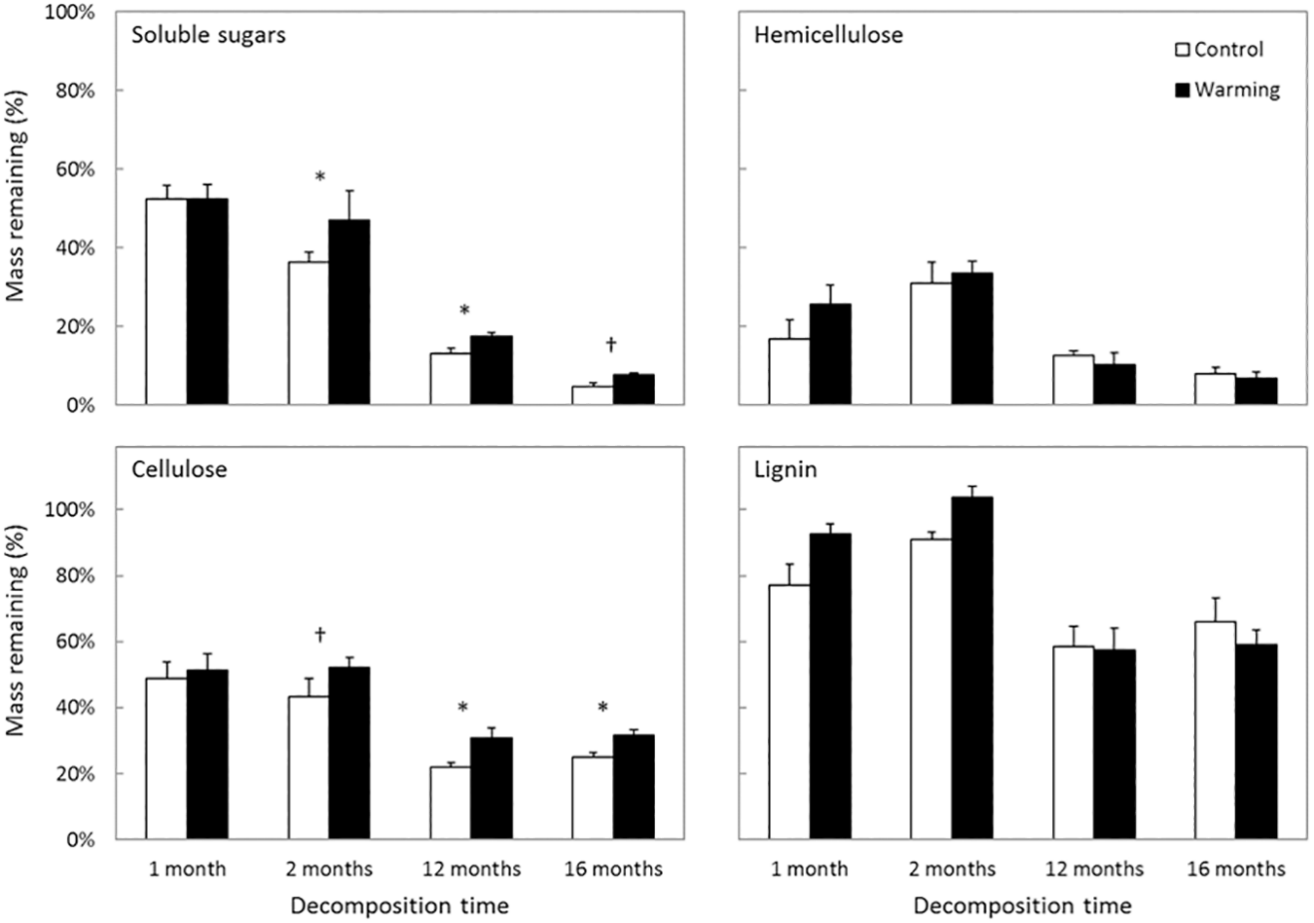
Percentage of mass remaining of lignin, cellulose, hemicellulose, and soluble sugar in spruce needles over time. Warming did not significantly affect lignin breakdown (P = 0.108) or hemicellulose breakdown (P = 0.179). In contrast, warming slowed the breakdown of cellulose significantly (P = 0.019) and soluble sugars marginally significantly (P = 0.077). Data are means ± SE, with n = 5 plots. *P < 0.05, †P < 0.10 for sampling date.

**Fig 4:**
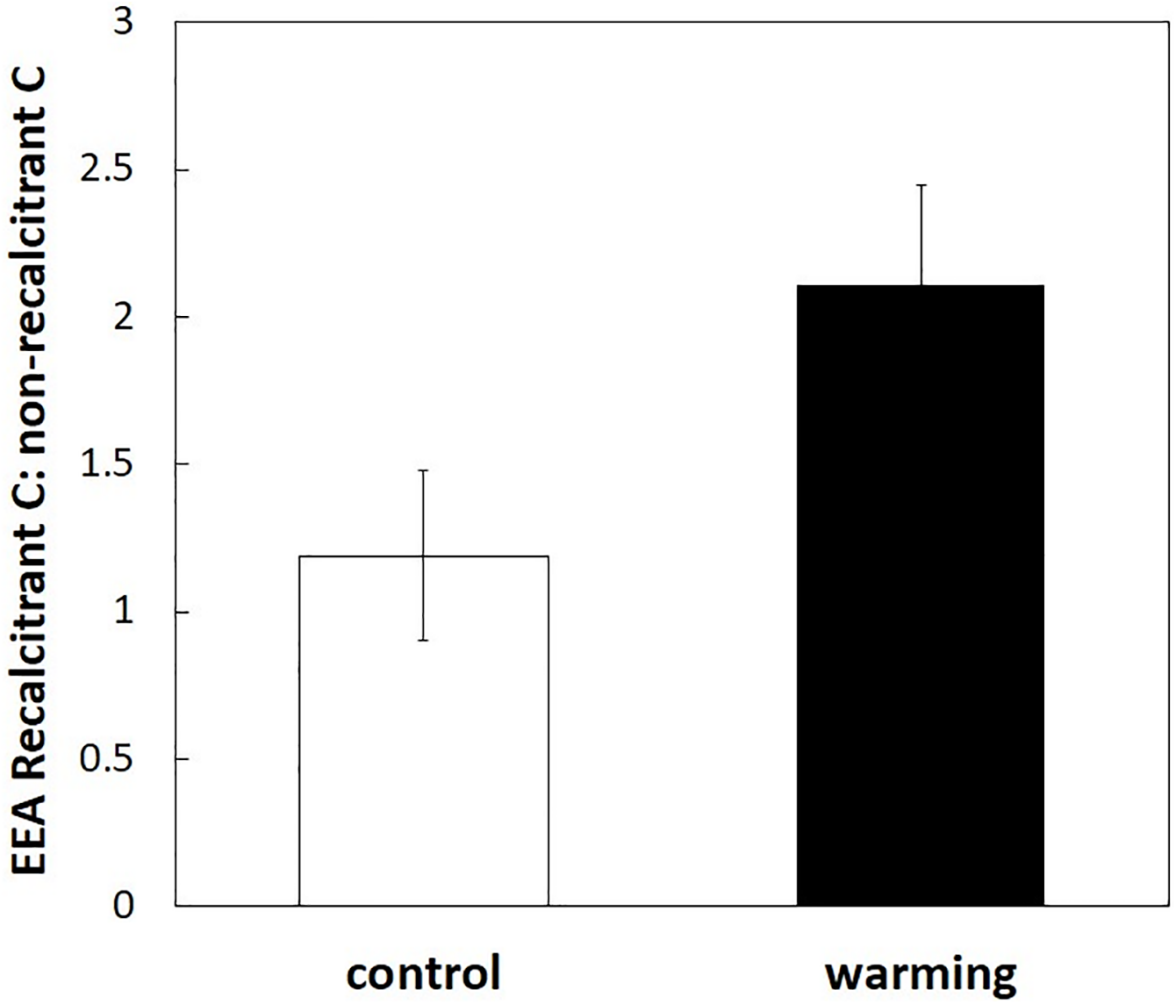
Extracellular enzyme activity (EEA) ratios of recalcitrant to non-recalcitrant C on litter retrieved at 12 months. Recalcitrant enzyme activity is polyphenol oxidase, while non-recalcitrant enzymes are the sum of cellobiohydrolase, β-xylosidase, and β-glucosidase. Warming significantly increased the ratio of recalcitrant C decay enzymes to non-recalcitrant C decay enzymes (P = 0.002). Activities were measured in units of nmol h^-1^ g^-1^ dry litter. Data are means ± SE, with n = 5 plots.

The warming effect on recalcitrant versus non-recalcitrant C after 12 months was attributable to declines in the breakdown of cellulose and soluble sugars, but not lignin (Fig 3, S1 Table and S1 Appendix). Warming did not significantly alter lignin loss across sampling dates (F_1,8_ = 3.28, P = 0.108), but cellulose loss was significantly slower in the warmed plots than in the control plots (F_1,8_ = 8.55, P = 0.019). In addition, soluble sugar loss was marginally reduced by warming (F_1,8_ = 4.121, P = 0.077). Hemicellulose loss was not significantly altered by warming (F_1,8_ = 2.17, P = 0.179). There were no significant interactions between sampling date and treatment for any of the chemical fractions (F_3,24_ < 1.89, P > 0.159 for all). Moreover, warming nearly doubled the ratio of recalcitrant C-targeting enzymes (i.e., PPO) to non-recalcitrant C-targeting enzymes (i.e., sum of BG, CBH, and BX) (Fig 4) (F_1,4_ = 46.86, P = 0.002). Most of this change was driven by the non-recalcitrant enzymes whose normalized activities declined from 1.29±0.11 to 0.86±21 (mean±SE) with warming. In contrast, normalized recalcitrant enzyme activity remained similar (1.05±0.15 for control versus 0.94±0.22 with warming).

## Discussion

In our study, we found that warming affected degradation of recalcitrant C versus non-recalcitrant C. Specifically, ratios of recalcitrant to non-recalcitrant C remaining were lower in the warming treatment compared to controls (Fig 1) despite slower overall litter decay (Fig 2). This shift in C decay ratios occurred because decay of non-recalcitrant C declined significantly with warming, but decay of recalcitrant C did not (Fig 3). Moreover, under warming, microbes shifted their allocation away from extracellular enzymes that targeted non-recalcitrant C (Fig 4). Altogether, we accepted our hypothesis that warming would reduce the ratio of recalcitrant C to non-recalcitrant C remaining in decomposed litter, consistent with a potential direct kinetic effect of warming and/or a shift in the fungal community with increased ability to break down recalcitrant C, as previously reported [17]. However, indirect warming effects like drying might have also influenced our results. Below we will discuss this possibility.

Moisture is a major control over decomposition in cold biomes [5,35–39]. Indeed, moisture constraints might have played an important role in decay dynamics in our experiment. Our warmed plots are on average 22% drier than control plots [24]. This drying effect likely contributed to declines in microbial biomass and soil respiration documented earlier in this field experiment [24]. In fact, this drying effect might be responsible for the overall slower decomposition in warmed plots compared to controls (Fig 2). Another abiotic factor that might be exerting control over decomposition is nutrient availability. For example, increases of nitrogen in soils can reduce fungal diversity [40] and biomass of microbial decomposers [41]. In a previous study in our experimental warming plots, warmed plots had a slight increase in nitrogen availability compared to control plots [24].

In addition to the indirect effect of drying on decomposition, we may have observed a direct warming effect. We found two lines of evidence for this effect. The first is the decline in recalcitrant: non-recalcitrant C ratios after 12 months of decomposition (Fig 1). The second is the shift in EEA away from enzymes that break down non-recalcitrant C (Fig 4). These responses are consistent with theory based on thermodynamics of chemical reactions—recalcitrant C is expected to be more temperature sensitive than non-recalcitrant C [3,11]. Even though microbial activity declined in general, warmer temperatures could have allowed those microbes that were active to better acquire energy from recalcitrant C. In addition, warming may have selected for microbial taxa that produce fewer non-recalcitrant-degrading enzymes because the resource returns from these enzymes were relatively lower under warming. However, this could be the effect of warming-induced drying. Previous research has shown that EEA of non-recalcitrant degrading enzymes (i.e. carbohydrate-degrading enzymes) decreases up to 63% with drying [42].

Treseder et al. [17] examined the fungal community composition in litterbags from the current study. They reported that eight years of experimental warming had selected for recalcitrant C-decomposers, mostly represented by free-living filamentous fungi. This finding mirrors earlier observations by McGuire et al. [43], who found that the ability to use lignocellulose was positively related to warming responses of fungal taxa after one year of warming in this experiment. A warming experiment in Harvard Forest documented similar results with bacteria, where the warming treatment tended to enrich putatively lignin-using bacterial taxa [15]. These previous studies suggest that the relative increase in recalcitrant C degradation that we found in our current study might be facilitated by community shifts toward microbial taxa with the capacity to enzymatically access and use these compounds.

If losses of recalcitrant C in litter increase under warming, what are the potential consequences for soil C storage? Decades- and century-old carbon is more temperature sensitive than months- and years-old carbon [44]. In this sense, most soil organic matter in high latitude ecosystems is considered recalcitrant since it is decades old or older [45]. Where soil moisture does not become more limiting with warming, an increase in recalcitrant C decay could reduce soil C storage. Nevertheless, the boreal forest we examined may not fit this scenario because warming-induced drying appeared to limit microbial activity, which could mitigate losses of soil C [46]. In our system, warming may serve to maintain the decomposition rates of recalcitrant C despite negative effects of moisture limitation on overall decomposition.

## Conclusion

In conclusion, our data suggest that in boreal ecosystems, recalcitrant C loss from litter differed in sensitivity to warming compared to non-recalcitrant C loss. Altogether, we found that warming decreased the ratio of recalcitrant to non-recalcitrant C, accompanied by a higher ratio of enzymes that target recalcitrant C. This change was consistent with previous observations of a shift in the fungal community toward lignin users. We stress the need to incorporate empirical measurements of recalcitrant C losses into field warming manipulations, along with assessments of microbial physiology like extracellular enzyme activities, to better assess the fate of litter inputs under warming in the next century.

## Supporting information

S1 Table Litter chemistry in litter decomposed in control and warmed plots. (PDF)

S1 Appendix Mass loss of each carbon fraction per plot. (XLSX)
