# Supplementary material for: Decomposition of recalcitrant carbon under experimental warming in boreal forest
Source: PLoS One. 2017 Jun 16;12(6):e0179674. doi: 10.1371/journal.pone.0179674 (PMC5473569; doi:10.1371/journal.pone.0179674)
Supplement: S1 Table — (PDF) [file pone.0179674.s001.pdf]

C fraction	Sampling time (months)	Control (mg)	Warmed (mg)
Lignin	Initial	287 ±15	
	1	221 ±18	265 ±9
	2	261 ±6	297 ±9
	12	168 ±17	164 ±19
	16	189 ±21	170 ±12
Cellulose	Initial	312 ±14	
	1	153 ±17	160 ±9
	2	135 ±4	163 ±10†
	12	69 ±5	95 ±5*
	16	78 ±6	99 ±6*
Hemicellulose	Initial	115 ±8	
	1	19 ±2	29 ±3
	2	36 ±3	39 ±3
	12	14 ±2	12 ±3
	16	9 ±2	8 ±1
Soluble sugars	Initial	21 ±1	
	1	11 ±1	11 ±2
	2	7 ±0	10 ±0*
	12	3 ±0	4 ±0*
	16	1 ±0	2 ±0†

Values are mean ±SE, n = 5 plots. *$P < 0.05$, †$P < 0.10$ between treatments.
